# Age-specific impact of different immunosuppressive regimens on kidney transplant outcomes: clinical evidence from the scientific registry of transplant recipients

**DOI:** 10.3389/fneph.2026.1886887

**Published:** 2026-08-14

**Authors:** Lina Maria Serna-Higuita, Sarah Kalmbach, Anja Schork, Nils Heyne, Sandra Beer-Hammer, Silvio Nadalin, Peter Martus, Alfred Königsrainer, Moritz Schmelzle, Markus Quante

**Affiliations:** 1 Institute of Clinical Epidemiology and Applied Biostatistics, University Hospital Tübingen, Tübingen, Germany; 2 Department of General, Visceral and Transplant Surgery, University Hospital Tübingen, Tübingen, Germany; 3 Department of Internal Medicine IV, Division of Diabetology, Endocrinology Nephrology, University Hospital Tübingen, Tübingen, Germany; 4 Department of Pharmacology, Experimental Therapy & Toxicology and Interfaculty Center of Pharmacogenomics & Drug Research (IZePhA), University Hospitals and Clinics, Eberhard Karls University Tübingen, Tübingen, Germany; 5 German Center for Diabetes Research (DZD), Tübingen, Germany; 6 Department of General, Visceral and Transplant Surgery, Hannover Medical School, Hannover, Germany

**Keywords:** age, graft loss, immunosuppressive regimens, kidney transplantation, survival

## Abstract

**Background:**

Although older kidney transplant recipients have a higher risk of side effects leading to infection and malignancies, the impact of different immunosuppressive regimens on clinical outcomes in this group remains unclear.

**Methods:**

Retrospective cohort study analyzing data from the Scientific Registry of Transplant Recipients database. Kidney transplant recipients were categorized according to age categories (18–64 and >65 years) and immunosuppressive maintenance regimens: tacrolimus/mycophenolate mofetil (Tac-MMF), Tac/mTOR-inhibitor (Tac-mTORi), cyclosporin/MMF (CsA-MMF) and mTORi-MMF. The primary endpoint was overall survival.

**Results:**

Among the 112, 952 patients analyzed, overall survival rates at 2-, 3- and 5-years post-transplant were 95.6%, 93.7% and 87.6% respectively. No significant differences in the risk of mortality, graft loss or acute rejection were observed between patients treated with Tac-mTORi and those receiving Tac-MMF. Furthermore, no significant interactions were found between the maintenance immunosuppressive regimens and recipient age groups. Regarding kidney function, younger patients on the Tac-mTORi compared with Tac-MMF regimen exhibited a lower mean eGFR throughout the follow-up period compared to those on Tac-MMF. In contrast to this younger cohort, older patients treated with Tac-mTORi did not experience a significant additional decline in renal function.

**Conclusion:**

In this study, Tac-mTORi and Tac-MMF showed comparable results regarding patient survival, graft survival and rejection rates. Because older patients remain at higher risk for drug-related side effects, including infections, metabolic disorders and malignancies, these results highlight the critical need for individually immunosuppressive regimens in older transplant recipients and warrant further prospective validation.

## What was known

- Older kidney transplant recipients have increased susceptibility to infections, malignancies, and metabolic complications related to immunosuppression, but are underrepresented in clinical trials.
- Evidence comparing long-term outcomes of different immunosuppressive regimens in older recipients is limited and inconsistent.

## This study adds

- In recipients ≥65 years, cumulative incidence of mortality was higher in patients on mTORi-MMF and CsA-MMF compared to Tac-MMF. In contrast, no increased mortality was observed in patients on Tac-mTORi compared to Tac-MMF.
- Tac–mTORi regimen showed comparable cumulative incidence of graft loss, acute rejection rate and trajectory of mean eGFR when compared to standard Tac–MMF regimen in older recipients.

## Potential impact

- These findings support individualized immunosuppressive strategies for older kidney transplant recipients rather than uniform treatment approaches.
- Tac-mTORi regimen might represent an alternative to Tac-MMF in selected older patients balancing efficacy and tolerability.

## Introduction

Kidney transplantation (KTx) is the most effective treatment to improve survival and quality of life in patients with end-stage renal disease (1, 2). In this context, the older patient population is becoming increasingly important, as the number of kidney transplant recipients (KTRs) being 65 years of age or older continues to grow in the United States (3). Older candidates face lower transplantation rates and higher waitlist attrition due to illness or mortality (4). Nevertheless, appropriately selected older transplant candidates may still achieve a significant survival benefit from KTx compared to remaining on dialysis (5, 6). Notably, the risk profile of older KTRs differs from younger cohorts. While immunosenescence results in lower acute rejection (AR) rates, older patients face a higher burden of comorbidities and susceptibility to immunosuppressive therapy–related side effects (7, 8). Furthermore, geriatric syndromes such as frailty have emerged as important prognostic factors, as they are associated with increased mortality and adverse outcomes after transplantation (9, 10). Consequently, assessing biological rather than chronological age is increasingly recommended for individualized recipient evaluation.

Immunosuppressive maintenance regimens for KTRs have evolved significantly, reducing rejection rates but introducing new challenges, particularly for older patients at higher risk for infections, malignancies, and diabetes. In this context, meticulous patient selection has become increasingly important (4). Older KTRs have an impaired immune function, a state also known as immunosenescence (11, 12). Since clinical trials for immunosuppressants traditionally focus on patients under 65 years of age (13), specific evaluations of graft lost risk in older patients are mostly lacking. An adaptation of immunosuppressive protocols according to the recipient’s age has rarely taken place (14). This leads to a gap in the scientific literature, making it difficult to determine the optimal treatment for older KTRs. Thus, the aim of this study was to evaluate whether recipient age (> 65 vs 18–65 years) is impacting the efficacy and safety profiles of different immunosuppressive maintenance regimens.

## Materials and methods

### Study population

**Figure 1:**
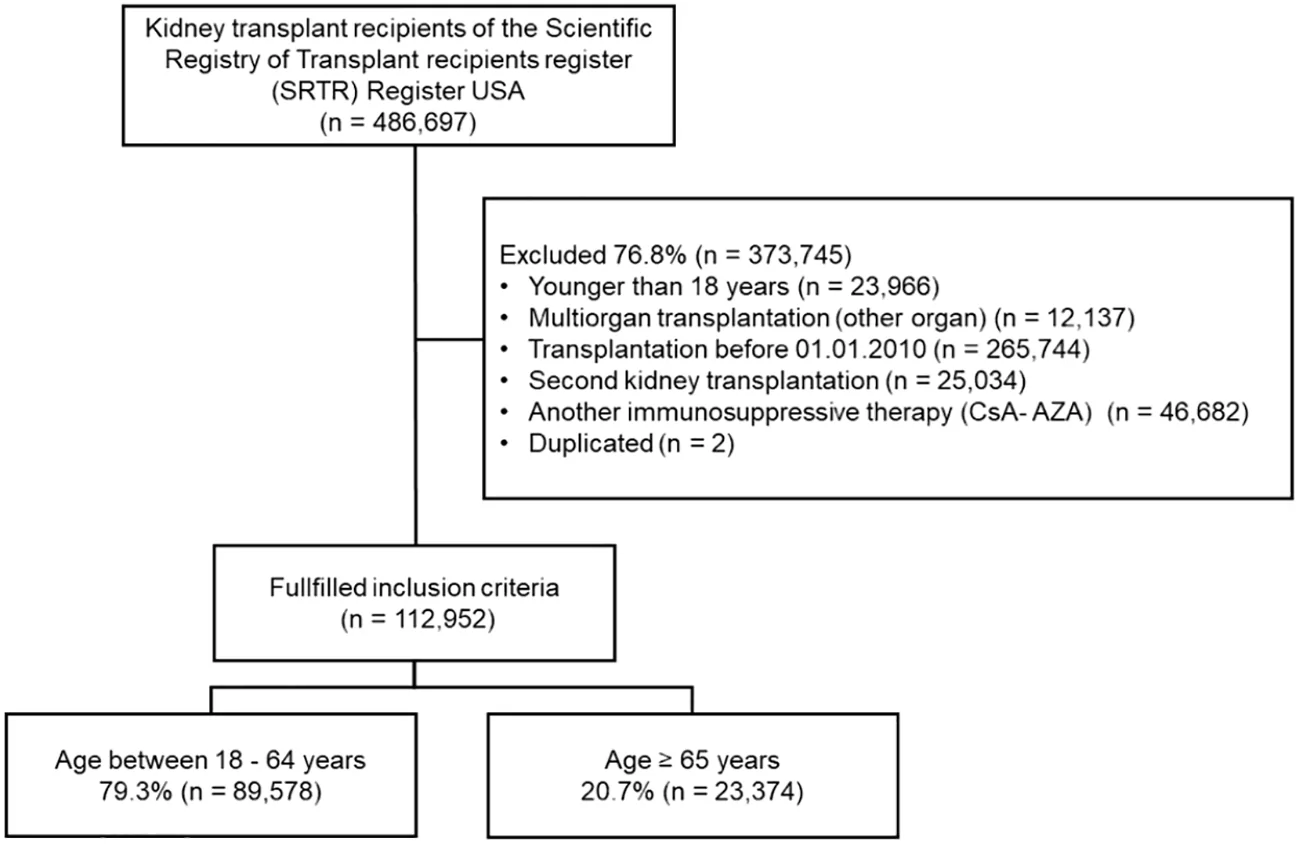
Flow chart.

This study used data from the Scientific Registry of Transplant Recipients (SRTR). The SRTR data system includes data on all donors, wait-listed candidates, and transplant recipients in the US, submitted by the members of the Organ Procurement and Transplantation Network (OPTN). The Health Resources and Services Administration (HRSA), U.S. Department of Health and Human Services provides oversight to the activities of the OPTN and SRTR contractors. Our study included adult patients (age ≥ 18 years), who underwent their first KTx between January 1, 2010, and December 31, 2020. Patients were excluded if they had received multi-organ transplants and had received immunosuppressive regimens other than the protocols of interest (**Figure 1**).

### Data collection

Baseline socio-demographic and clinical characteristics for both donors and recipients were recorded, including age at transplantation, sex, weight, BMI, and pre-existing comorbidities. Other variables collected were the number of human leukocyte antigen (HLA) mismatches, type of transplantation, induction and immunosuppressive maintenance regimens documented three months after KTx. Age was stratified in two groups (18–64 years and ≥ 65 years) based on the CDC (Centers for Disease Control and Prevention) classification of older adults (15). Immunosuppressive regimens were categorized into four groups: 1) Tac-MMF, 2) Tac-mTORi, 3) CsA-MMF and 4) mTORi-MMF. The analysis was restricted to these four dominant regimens to accurately reflect clinical practices during the study period. These regimens directly reflect international standard-of-care guidelines (16). Specifically, the KDIGO Clinical Practice Guidelines for the Care of Kidney Transplant Recipients recommend a calcineurin inhibitor (CNI; preferably Tacrolimus or CsA) as first-line therapy, combined with an antiproliferative agent (preferring Mycophenolate). KDIGO also recognizes mTOR inhibitors as important, tailored alternatives within maintenance strategies (17–19).

### Endpoint definition

The primary outcome was the cumulative incidence of overall survival (OS), defined as death for any cause. Patients alive or lost to follow-up were right censored at the time of the last registered contact. The maximum follow-up was 10 years. Secondary outcomes included cumulative incidence of cause-specific mortality, graft loss (GL), 1-year acute rejection (AR) and kidney function. Kidney function was calculated using the estimated glomerular filtration rate (eGFR) calculated with the 2021 Chronic Kidney Disease Epidemiology Collaboration (CKD-EPI) equation without race adjustment (20).

### Statistical analysis

Baseline recipient and donor characteristics were analyzed using descriptive statistics. Nominal variables were summarized using absolute counts and percentages. Continuous variables were reported as mean ± standard deviation (SD) or median (interquartile range). Data distribution was evaluated by investigating kurtosis, skewness, as Q-Q plots, and histograms.

The statistical analyses were conducted utilizing a landmark cohort design. For the primary endpoint OS, Kaplan-Meier methods were used to estimate post-transplant survival probabilities. Proportional Cox regression models were applied to evaluate the association between age and immunosuppressive regimens on the outcome OS. Exposure variable selection was based on clinical reasoning (1, 5, 21–25) and supported by two approaches: univariate analysis and Survival Random Forest model (26–28) (**Supplementary Figure 1**). These selected covariable were subsequently included in the final multivariable Cox regression model. To evaluate whether age modified the effect of immunosuppressive maintenance regimens on the outcomes, an interaction term between age and immunosuppressive regimens was included. Where a significant interaction was detected, subgroup analyses were performed and reported with corresponding p-values; in the absence of significant interaction, only the effect sizes for the subgroups were reported. For all comparative analyses, Tac-MMF group was designated as the reference group, against which the other three regimens (Tac-mTORi, CsA-MMF, and mTORi-MMF) were compared.

The Aalen-Johansen estimator was used to calculate the cumulative incidence of the secondary endpoint GL. Sub-distribution Cox Proportional-Hazard models of Fine and Gray were used to assess association between GL across age and immunosuppressive regimens. Death was considered as competing risk. The Schoenfeld test and the goodness of fit test based on the cumulative sum of residuals (29) were used to assess the proportionality assumption required for Cox proportional and Subdistribution hazard modeling.

1-year AR was described using absolute frequencies and percentages. Binary logistic regression was performed to compare age and immunosuppressive regimens; odds ratios (OR) and two-sided 95% CI were calculated. The goodness of fit of the model was evaluated using the Hosmer-Lemeshow test. The effect of age and immunosuppressive regimen on the eGFR slope was analyzed by fitting a linear mixed model with random intercepts for each participant over time. The model included fixed effects for treatment, age and the interactions term of treatment with time.

Statistical significance was assumed for two-sided values of 0.05 or less. All statistical analyses were performed using R version 4.1.0 and SPSS for Windows version 28 (IBM).

## Results

### Demographics

Out of the 486, 697 recipients recorded in the SRTR database between 2010 and 2020, a total of 112, 952 (43.1%) were included in the final analysis (**Figure 1**). Mean age at the time of KTx was 52.2 years (SD ±13.8), with 89, 578 patients aged 18–64 years and 23, 374 patients aged ≥ 65 years. Patients´ characteristics stratified according to the respective age categories are shown in **Table 1**. When compared to the younger group, older patients (>65 years) had a higher prevalence of angina, diabetes and malignancies. The maintenance immunosuppressive regimens used three months after KTx were n = 110, 866 (98.2%) Tac-MMF, n = 500 (0.4%) Tac-mTORi, n = 1, 241 (1.1%) CsA-MMF, and n = 345 (0.3%) mTORi-MMF. A total of 75, 155 (66.5%) patients received steroid maintenance regimen, with no difference between age groups.

**Table 1:** Baseline characteristics stratified by age.

Variable	Missing values	Age 18–64 years (n=89, 578)	Age ≥ 65 years (n=23, 374)	Total (N = 112, 952)	SMD
Recipient characteristics					
Gender	0				
female n (%)		35, 109 (39.2%)	8, 568 (36.7%)	43, 677 (38.7%)	0.052
Age at transplant	0				
mean (SD)		47.6 (± 11.8)	69.4 (± 3.68)	52.2 (± 13.8)	2.496
Weight (Kg)	0				
mean (SD)		83.2 (19.8)	81.8 (17.1)	82.9 (19.3)	0.076
Height (cm)	0				
mean (SD)		171 (10.8)	170 (10.4)	171 (10.7)	0.104
BMI	0				
mean (SD)		28.4 (5.57)	28.3 (4.82)	28.4 (5.43)	0.013
Dialysis before transplantation					
yes n (%)	184 (0.16%)	71, 740 (80.2%)	18, 273 (78.3%)	90, 013 (79.8%)	0.047
Previous Diabetes					
Yes n (%)	225 (0.20%)	27, 829 (31.1%)	11, 337 (48.6%)	39, 166 (34.7%)	0.363
Previous malignancy					
yes n (%)	792 (0.70%)	799 (0.9%)	551 (2.4%)	1, 350 (1.2%)	0.116
Time on wait list					
median (IQR)	1, 538 (1.36%)	14.0 [5, 36]	16.0 [5, 38]	15.0 [5, 36]	0.037
Number of HLA mismatches					
median (IQR)	622 (0.55%)	4 [3, 5]	4 [3, 5]	4 [3, 5]	0.041
Cold Ischemic Time					
median (IQR)	3, 192 (2.82%)	9 [1.3, 17.4]	12 [2.1, 20]	9.67 [1.4, 18]	0.213
Donor´s characteristics					
Age in years	0				
mean (SD)		40.2 (13.3)	47.6 (13.9)	41.7 (13.8)	0.545
Gender	0				
female n (%)		44, 245 (49.4%)	12, 016 (51.4%)	56, 261 (49.8%)	0.040
male n (%)		45, 333 (50.6%)	11, 358 (48.6%)	56, 691 (50.2%)	
Donor type	0				
living n (%)		38, 757 (43.3%)	7, 817 (33.4%)	46, 574 (41.2%)	0.203
deceased n (%)		50, 821 (56.7%)	15, 557 (66.6%)	66, 378 (58.8%)	
Deceased expanded criteria		6, 990 (7.8%)	5, 560 (23.8%)	12, 559 (11.1%)	
Creatinine > 1.5 mg/dl					
yes n (%)	349 (0.31%)	11, 353 (12.7%)	3, 964 (17.0%)	15, 317 (13.6%)	0.078
mmunosuppressive therapy used					
Induction	0				
Yes n (%)		83, 243 (92.9%)	21, 693 (92.8%)	104, 936 (92.9%)	0.005
Steroids induction	0				
Yes n (%)		61, 722 (68.9%)	15, 792 (67.6%)	77, 514 (68.6%)	0.029
Steroid maintenance	0				
Yes n (%)		59, 460 (66.4%)	15, 695 (67.1%)	75, 155 (66.5%)	0.016
immunosuppressive regimen	0				
Tac-MMF		87, 944 (98.2%)	22, 922 (98.1%)	110, 866 (98.2%)	0.013
Tac-mTORi		403 (0.4%)	97 (0.4%)	500 (0.4%)	
CsA-MMF		962 (1.1%)	279 (1.2%)	1, 241 (1.1%)	
mTORi-MMF		269 (0.3%)	76 (0.3%)	345 (0.3%)	

SMD, Standardized mean differences.

### Overall survival

**Figure 2:**
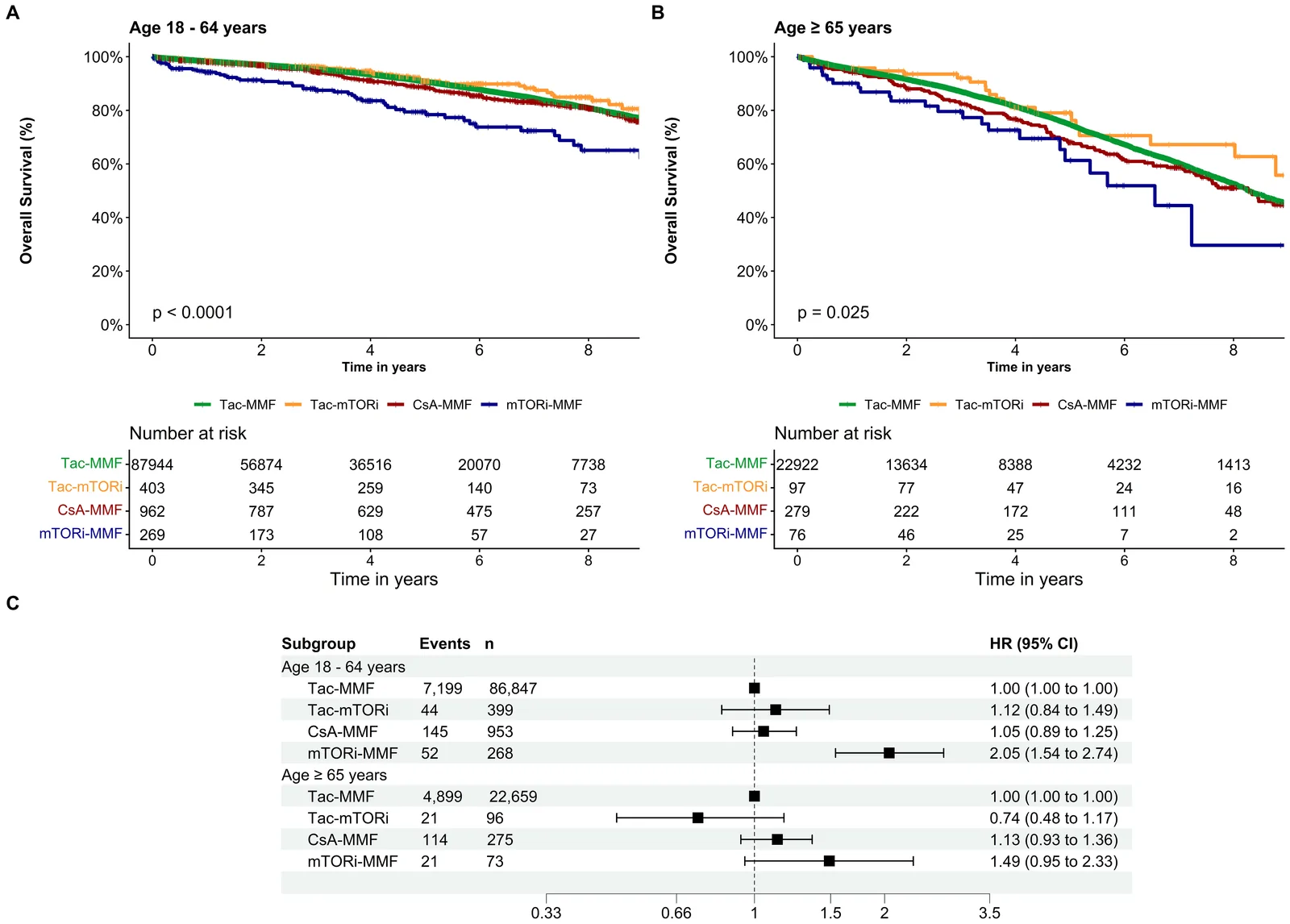
Overall survival stratified by maintenance immunosuppressive regimen and age. **(A, B)** Kaplan–Meier estimates of overall survival stratified by age group and maintenance immunosuppressive regimen. **(C)** Forest plot displaying subgroup analyses from Cox proportional hazards models for overall survival, stratified by age group. Multivariate model was adjusted for baseline recipient characteristics, including gender, BMI, pre-transplant dialysis status, pre-existing diabetes. Donor related variables including donor age and donor type (living, standard criteria deceased and expanded criteria deceased); and transplant-specific variables included: cold ischemia time, number of HLA mismatches, induction therapy and steroid maintenance therapy.

During a median follow-up of 3.67 years (95% CI: 3.61 to 3.72), a total of 12, 356 KTRs died. The OS was 95.6%, 93.7% and 87.6% at, 2-, 3- and 5-years post-transplant respectively (**Figures 2a, b**). The lowest OS was observed among KTRs age ≥65 years who received CsA-MMF and mTORi-MMF (see **Supplementary Table **). Results of the univariate analyses are shown in **Supplementary Table 2**. In multivariable analysis, patients receiving mTORi-MMF tended to a higher risk of all-cause mortality (aHR 1.46 95% CI 0.93 – 2.29 p-value = 0.099) compared with those receiving Tac-MMF. By contrast, the Tac-mTORi group was not associated with an increased risk of death (aHR 0.74, p-value 0.205) (**Table 2**). The effect of immunosuppressive regimens on OS was consistent across age subgroups, with no significant interaction per subgroup (p-value_interaction_ Tac-mTORi: 0.166, CsA-MMF: 0.658 and mTORi-MMF: 0.197). **Figure 2c** shows the subgroup analysis, indicating that the Tac-mTORi and Tac-MMF regimens were associated with comparable OS.

**Table 2:** Multivariable analysis of the endpoint all-cause of death, proportional hazard ratio model (n = 111, 570).

Variable	HR	95% CI	P value
Age			
≥ 65 years	1		
18–64 years	0.43	0.41 – 0.44	<0.001
Immunotherapy			
Tac-MMF	1		
Tac-mTORi	0.74	0.47 – 1.17	0.205
CsA-MMF	1.12	0.92 – 1.36	0.265
mTORi-MMF	1.46	0.93 – 2.29	0.099
Interaction term			
Tac-mTORi: Age 18–64	1.46	0.85– 2.51	0.166
CsA-MMF: Age 18–64	0.94	0.73 – 1.22	0.658
mTORi-MMF: Age 18–64	1.42	0.83 – 2.41	0.197

Multivariate model was adjusted for baseline recipient characteristics, including gender, BMI, pre-transplant dialysis status, pre-existing diabetes. Donor related variables including donor age and donor type (living, standard criteria deceased and expanded criteria deceased); and transplant-specific variables included: cold ischemia time, number of HLA mismatches, induction therapy and steroid maintenance therapy.

### Cause-specific death

**Figure 3:**
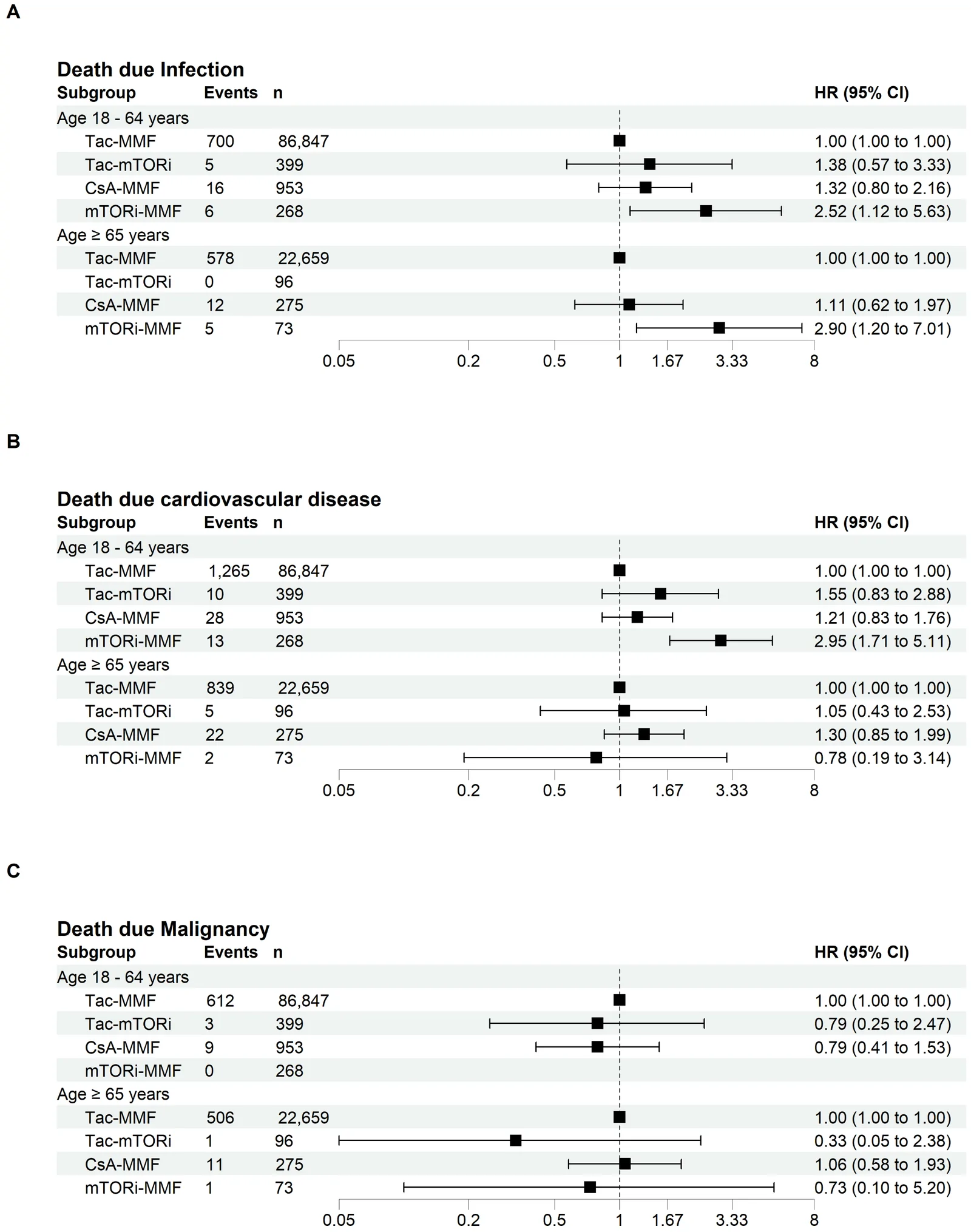
Forest plot of multivariate Cox proportional hazards models for cause-specific mortality stratified by age: **(A)** death due to infections, **(B)** death due to cardiovascular disease, and **(C)** death due to malignancy. Tac-MMF serves as the reference category across all comparisons. All models were adjusted for baseline recipient characteristics (gender, BMI, pre-transplant dialysis status, and pre-existing diabetes), donor-related variables (donor age and donor type [living, standard criteria deceased, or expanded criteria deceased]), and transplant-specific variables (cold ischemia time, number of HLA mismatches, induction therapy, and steroid maintenance therapy). Abbreviations: CI, confidence interval; CsA, cyclosporine A; HLA, human leukocyte antigen; HR, hazard ratio; MMF, mycophenolate mofetil; mTORi, mammalian target of rapamycin inhibitor; Tac, tacrolimus.

Hazard ratios for cause-specific death are presented in **Table 3**. Treatment with Tac-mTORi was not associated with an increased risk of cause-specific mortality compared with Tac-MMF. In contrast, KTRs receiving mTORi-MMF demonstrated a higher risk of death from infection (HR 2.64, p value <0.001). Subgroup analyses confirmed that this association was consistent across prespecified age groups (**Figure 3**).

**Table 3:** Proportional hazard ratio model of cause specific death (n = 111, 750).

Variable	HR	95% CI	P value
Death due infection			
Age			
≥ 65 years	1		
18–64 years	0.38	0.34 – 0.43	<0.001
Immunotherapy			
Tac-MMF	1		
Tac-mTORi	0.71	0.30 – 1.72	0.452
CsA-MMF	1.21	0.83 – 1.77	0.311
mTORi-MMF	2.64	1.46 – 4.78	0.001
Death due cardiovascular disease			
Age			
≥ 65 years			
18–64 years	0.46	0.42 – 0.50	<0.001
Immunotherapy			
Tac-MMF	1		
Tac-mTORi	1.32	0.79 – 2.19	0.283
CsA-MMF	1.25	0.94 – 1.65	0.124
mTORi-MMF	2.14	1.29 – 3.56	0.003
Death due malignancy			
Age			
≥ 65 years	1		
18–64 years	0.30	0.27 – 0.34	<0.001
Immunotherapy			
Tac-MMF	1		
Tac-mTORi	0.58	0.22 – 1.56	0.283
CsA-MMF	0.92	0.59 – 1.43	0.701
mTORi-MMF	0.29	0.04 – 2.05	0.214

Multivariate model was adjusted for baseline recipient characteristics, including gender, BMI, pre-transplant dialysis status, pre-existing diabetes. Donor related variables including donor age and donor type (living, standard criteria deceased and expanded criteria deceased); and transplant-specific variables included: cold ischemia time, number of HLA mismatches, induction therapy and steroid maintenance therapy.

### Graft loss

**Figure 4:**
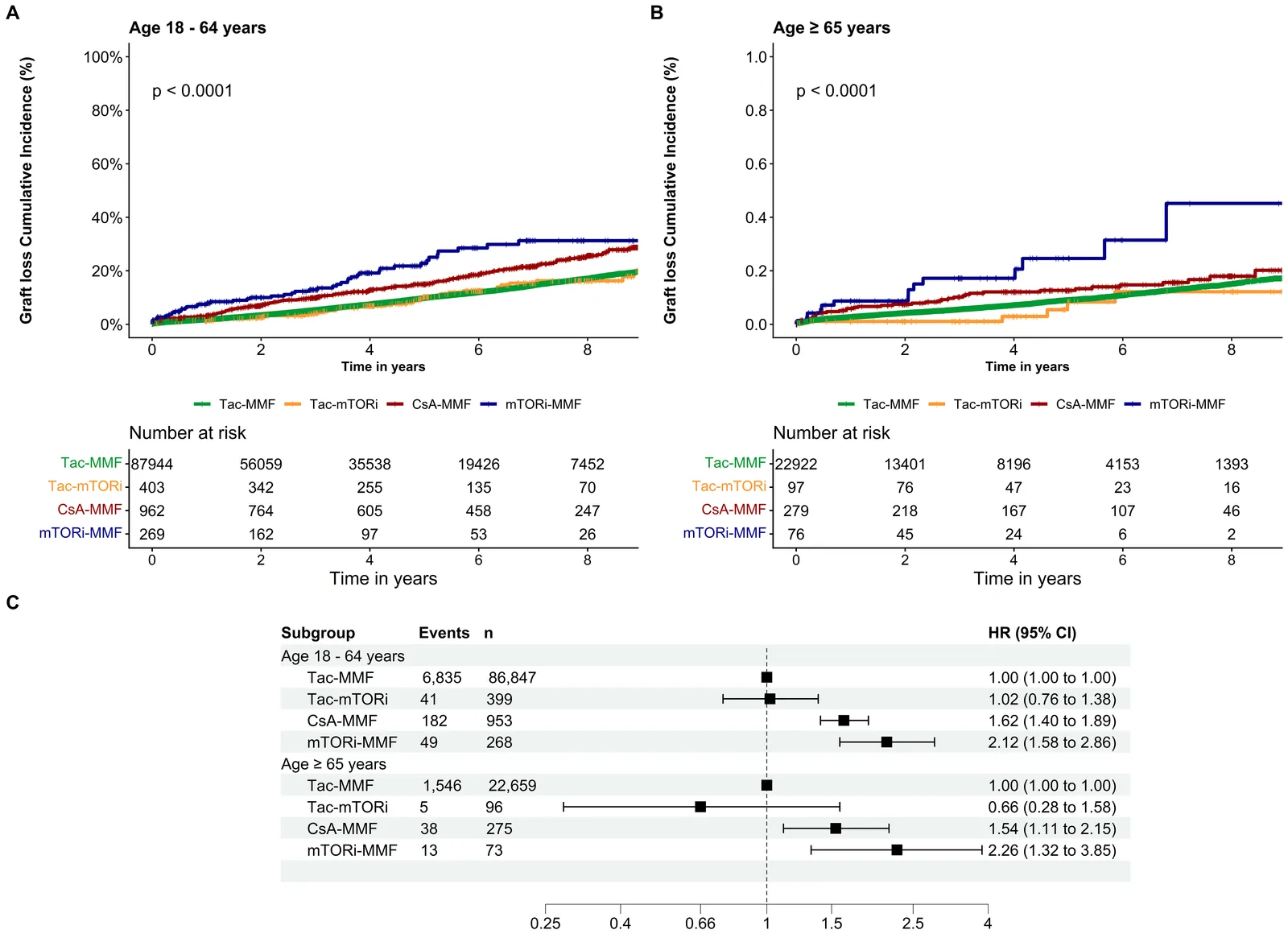
Cumulative incidence and subdistribution fine and gray model of graft loss stratified by maintenance immunosuppressive regimen and age. **(A, B)** Cumulative incidence of graft loss stratified by age group and maintenance immunosuppressive regimen. **(C)** Forest plot displaying subgroup analyses from subdistribution Fine and Gray models for graft loss, stratified by age group. Multivariate model was adjusted for baseline recipient characteristics, including gender, BMI, pre-transplant dialysis status, pre-existing diabetes. Donor related variables including donor age and donor type (living, standard criteria deceased and expanded criteria deceased); and transplant-specific variables included: cold ischemia time, number of HLA mismatches, induction therapy and steroid maintenance therapy.

GL was observed in 8, 654 out of 112, 952 patients, while 10, 080 patients died with a functioning graft. Cumulative incidences of death censored GL are show in **Figures 4a, b**. **Supplementary Table 3**, **Supplementary Figure 2** show the cumulative incidence of GL and death with functioning graft across therapies and age groups. GL rates varied strongly between the different therapies and age categories, being highest in the older mTORi-MMF group. Two-year cumulative incidence of GL in patients ≥ 65 years was lowest with 1.0% in patients with Tac-mTORi and increased to 4.1% in patient with Tac-MMF and up to 8.4% in patients receiving mTORi-MMF (**Supplementary Table 3**).

In the multivariable subdistribution hazard ratio (sHR) analysis, the risk of graft loss (GL) was significantly higher in patients receiving CsA-MMF (sHR 1.65, 95% CI 1.42 – 1.92) and mTORi-MMF (sHR 2.11 95% CI 1.57 – 2.84) compared to patients with Tac-MMF (**Table 4**). By contrast, no significant difference in the risk of GL was observed for KTRs treated with Tac-mTORi (sHR 1.04 95% CI 0.77 – 1.41 p-value = 0.780). This association remained consistent by age groups (p-value_interaction_ CsA-MMF: 0.430, Tac-mTORi: 0.290 and mTORi-MMF: 0.670). The effect of the immunosuppressive regimens on GL was further evaluated across the two age groups. Here age did not appear to modify the effect of immunosuppressive regimen on GL (**Figure 4c**).

**Table 4:** Subdistribution hazard ratio model (Fine and Gray) of graft loss, death is considered as competing risk (n = 111, 570).

Variable	sHR	95% CI	P value
Age			
≥ 65 years	1		
18–64 years	1.35	1.27 – 1.42	<0.001
Immunotherapy			
Tac-MMF	1		
Tac-mTORi	1.04	0.77 – 1.41	0.780
CsA-MMF	1.65	1.42 – 1.92	<0.001
mTORi-MMF	2.11	1.57 – 2.84	<0.001
Interaction term			
Tac-mTORi: Age 18–64	1.65	0.66 – 4.14	0.290
CsA-MMF: Age 18–64	1.16	0.80 – 1.67	0.430
mTORi-MMF: Age 18–64	0.87	0.46 – 1.64	0.670

Multivariate model was adjusted for baseline recipient characteristics, including gender, BMI, pre-transplant dialysis status, pre-existing diabetes. Donor related variables including donor age and donor type (living, standard criteria deceased and expanded criteria deceased); and transplant-specific variables included: cold ischemia time, number of HLA mismatches, induction therapy and steroid maintenance therapy.

### Acute rejection during the first year after KTx

**Figure 5:**
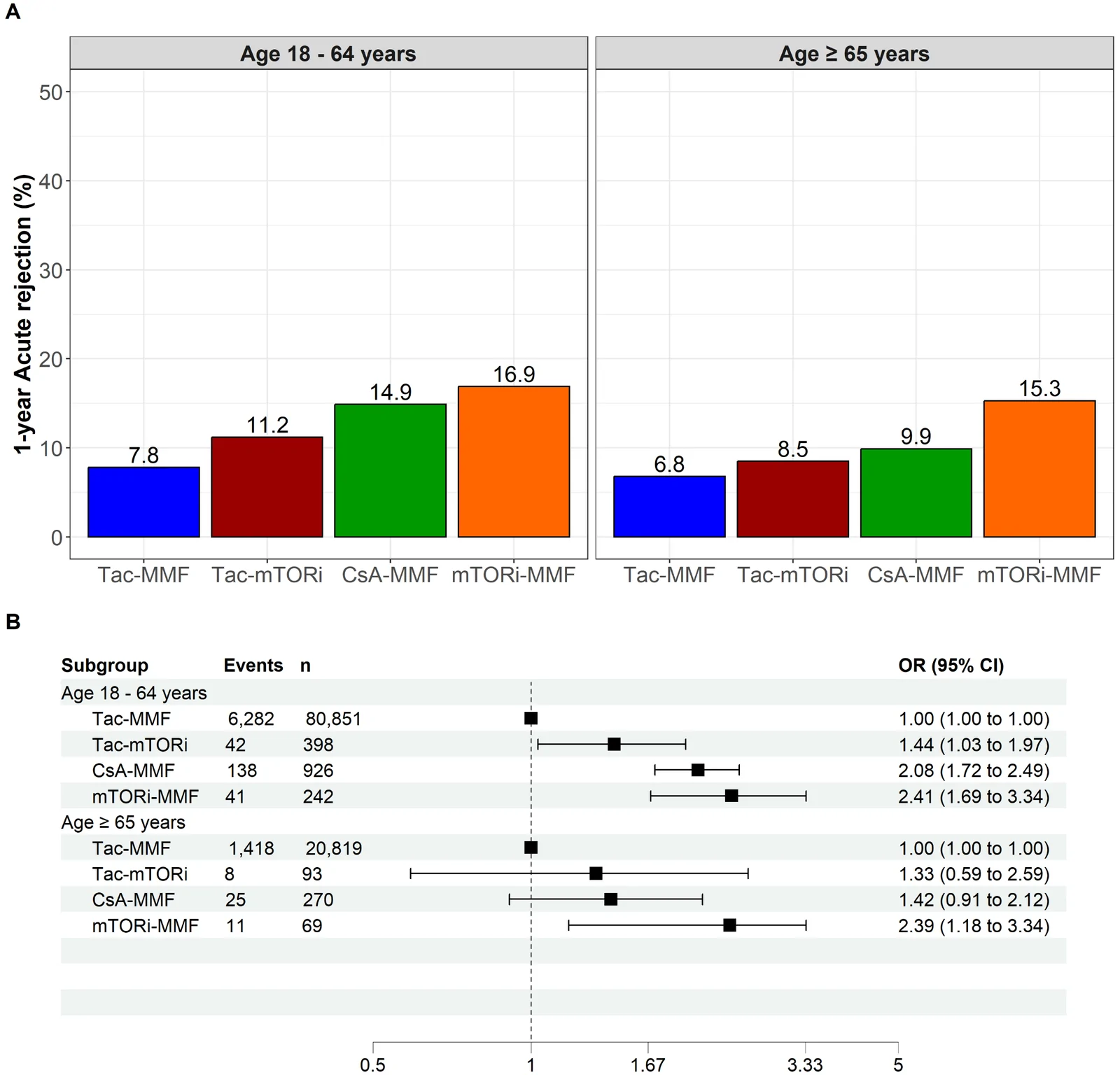
Proportion of 1-year AR stratified by age and immunosuppressive regimen. **(A)** Proporcion of acute rejection stratified by age group and immunosuppressive regimen. **(B)** Forest plot displaying subgroup analyses from binary logistic regression models for acute rejection, stratified by age group. Multivariate model was adjusted for baseline recipient characteristics, including gender, BMI, pre-transplant dialysis status, pre-existing diabetes. Donor related variables including donor age and donor type (living, standard criteria deceased and expanded criteria deceased); and transplant-specific variables included: cold ischemia time, number of HLA mismatches, induction therapy and steroid maintenance therapy.

Episodes of 1-year AR occurred in 7.7% of the total study cohort (8, 062/104, 788). **Supplementary Table 4** and **Figure 5a** show the percentages of 1-year AR episodes stratified by immunosuppressive regimens and age categories. Here, mTORi-MMF was associated with highest incidences of acute rejection across both age categories, reaching the highest value in patients younger than 65 years (16.9%). By contrast, the lowest incidence of 1-year AR was observed in young patients when treated with Tac-MMF and in the older cohort upon treatment with Tac-mTORi. Multivariable binary logistic regression model was performed to assess the association between age, immunosuppressive regimens, and the risk of 1-year AR. KTRs of 18–64 years had a higher likelihood of rejection (OR 1.21 95% CI 1.13 – 1.28). Treatment with mTORi-MMF was associated with an increased risk of AR (OR: 2.39 95% CI 1.18 – 4.40), whereas Tac-mTORi was not significantly different from Tac-MMF (OR: 1.31 95% CI 0.58 – 2.55). Interaction terms between age and therapy were not statistically significant, suggesting that the effect of immunosuppressive regimens on AR episodes did not differ across age groups (**Table 5**). The effect of the immunosuppressive regimens on the endpoint 1-year AR was further evaluated across the two age groups. Here, age did not impact the effect of immunosuppressive regimens on 1-year AR (**Figure 5b**).

**Table 5:** Binary logistic regression model of acute rejection (n = 103, 668).

Variable	OR	95% CI	P value
Age			
≥ 65 years	1		
18–64 years	1.21	1.13 – 1.28	<0.001
Immunotherapy			
Tac-MMF	1		
Tac-mTORi	1.31	0.58 – 2.55	0.467
CsA-MMF	1.47	0.95 – 2.18	0.070
mTORi-MMF	2.39	1.18 – 4.40	0.009
Interaction term			
Tac-mTORi: Age 18–64	1.10	0.52 – 2.63	0.805
CsA-MMF: Age 18–64	1.41	0.91 – 2.27	0.139
mTORi-MMF: Age 18–64	1.003	0.49 – 2.18	0.992

Multivariate model was adjusted for baseline recipient characteristics, including gender, BMI, pre-transplant dialysis status, pre-existing diabetes. Donor related variables including donor age and donor type (living, standard criteria deceased and expanded criteria deceased); and transplant-specific variables included: cold ischemia time, number of HLA mismatches, induction therapy and steroid maintenance therapy.

### Kidney function

**Figure 6:**
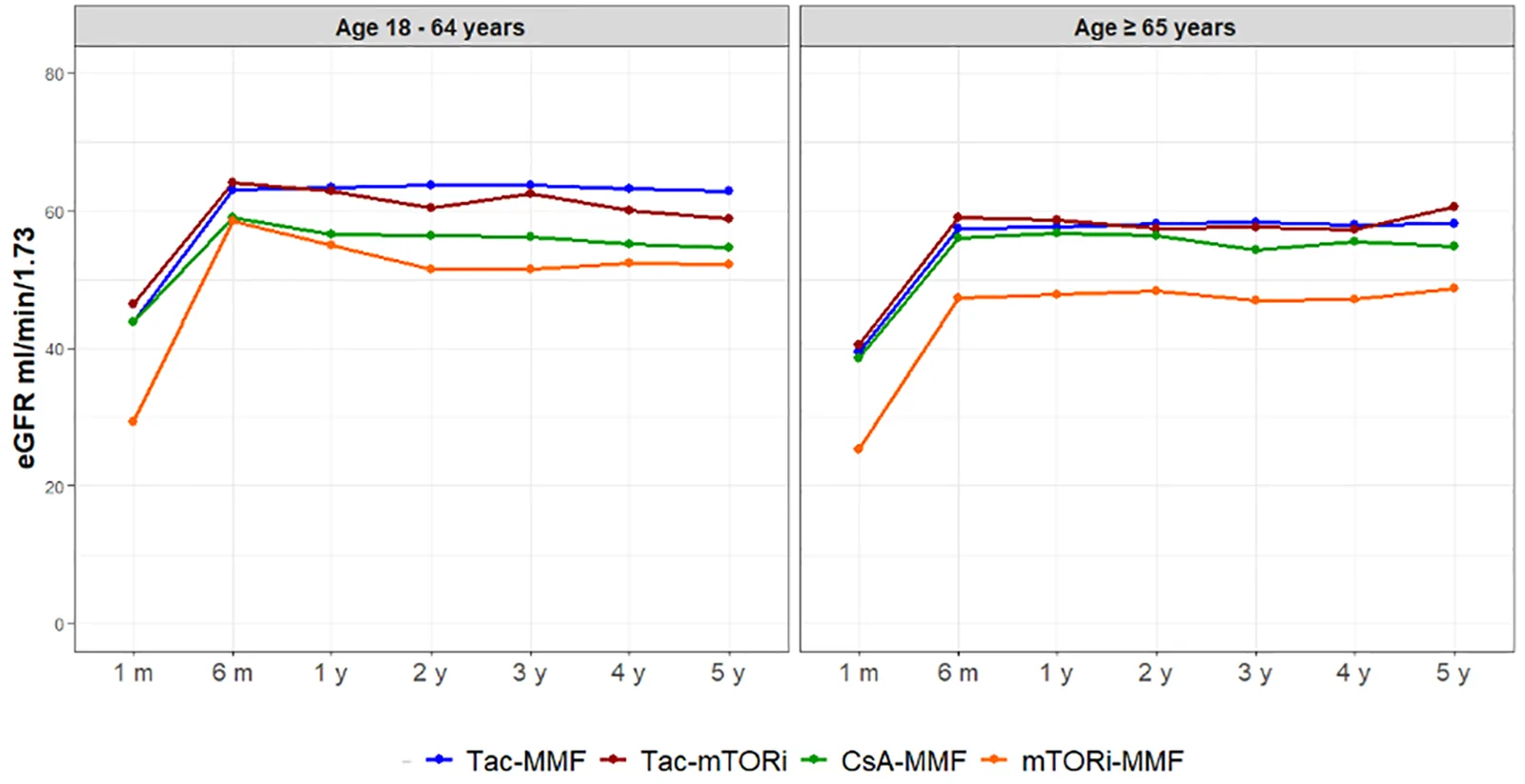
Longitudinal kidney function up to 5 years post-transplantation mean values of eGFR during the first 5 years after kidney transplantation, stratified by age groups and immunosuppressive regimen. eGFR calculated by CKD EPI without race.

**Figure 6** and **Supplementary Table 5** present the trajectory of mean estimated glomerular filtration rate (eGFR) over the first five years after KTx, stratified by age group and immunosuppressive maintenance regimen. Among KTRs of 18–64 years, initial kidney function one month after KTx was significantly reduced in the mTORi-MMF group compared with Tac-MMF, with an estimated effect size of β = –7.719 [mean eGFR = 75.36], p-value < 0.001). A similar pattern was observed among older KTRs (≥65 years), where baseline eGFR was also lowest in the mTORi-MMF group (β = –9.985, [mean eGFR = 67.67, p-value < 0.001) (**Table 6**). No differences were observed in patients older than 65 years receiving Tac-mTORi or CsA-MMF.

**Table 6:** Linear mixed model of changes of mean eGFR over time (in years).

Variable	β	95% CI	P value
Cohort 18–64 years			
Intercept	83.08	82.124 – 84.032	
Time	-0.234	-0.263 – -0.204	<0.001
Immunotherapy			
Tac-MMF			
Tac-mTORi	0.030	-0.276 – 2.135	0.978
CsA-MMF	-5.295	-6.689 – -3.901	<0.001
mTORi-MMF	-7.719	-10.515 – -4.923	<0.001
Interaction term			
Tac-mTORi: Time	-0.971	-1.342 – -0.601	<0.001
CsA-MMF: Time	-0.674	-0.922 – -0.426	<0.001
mTORi-MMF: Time	-1.045	-1.612 – -0.477	<0.001
Cohort ≥ 65 years			
Intercept	77.66	75.70 – 79.61	
Time	-0.309	-0.365 – -0.254	<0.001
Immunotherapy			
Tac-MMF			
Tac-mTORi	-0.059	-4.102 – 3.984	0.977
CsA-MMF	-1.265	-3.674 – 1.143	0.303
mTORi-MMF	-9.985	-14.944 – -5.026	<0.001
Interaction term			
Tac-mTORi: Time	0.087	-0.678 – 0.852	0.824
CsA-MMF: Time	-0.508	-0.930 - -0.087	0.018
mTORi-MMF: Time	0.191	-0.824 – 1.205	0.712

Adjusted for BMI, type of donor, induction therapy, steroid maintenance therapy, cold ischemic time and HLA mismatches.

Longitudinal trajectories of eGFR revealed age-specific differences. In the younger cohort (18–64 years), the mean annual decline in eGFR for patients receiving the Tac-MMF regimen was –0.234 mL/min/1.73 m² per year, representing the slowest rate of decline in this age group. In contrast, patients treated with other regimens exhibited significantly greater annual reductions: CsA-MMF (β = –0.674), Tac-mTORi (β = –0.971), and mTORi-MMF (β = –1.045). In the older cohort (≥65 years), a different pattern was observed. The mean annual decline in eGFR among patients treated with Tac-MMF was –0.309 mL/min/1.73 m² per year, broadly comparable to the trajectory seen in younger KTRs. However, in contrast to the younger cohort, no significant additional decline was observed in patients treated with either Tac-mTORi (β = 0.087) or mTORi-MMF (β = 0.191) (**Figure 6**).

## Discussion

Although the number of KTRs being 65 years of age or older is steadily increasing in parallel with demographic changes in the general population (30, 31), we are still facing a substantial gap of knowledge on how different immunosuppressive regimens impact the clinical outcome after kidney transplantation in an age-specific fashion. To address this question, our study evaluated the age-stratified impact of different maintenance immunosuppressive regimens on post-transplant clinical outcomes. Our findings indicate that a Tac – mTORi based maintenance regimen may provide comparable therapeutic efficacy to Tac – MMF in terms of 1-year acute rejection, graft loss and overall survival, with no significant differences observed between patients aged 18–64 and those ≥ 65 years. These findings support the conclusion of Sanders et al. (32),, suggesting that both regimens may be effective options for older KTRs.

In detail, our analysis revealed a higher prevalence of angina, diabetes, and prior malignancy among older KTRs, consistent with previous literature (33, 34). Furthermore, the older cohort exhibited a significantly higher risk of mortality due to infection, cardiovascular disease and malignancies. Despite these elevated risks, our findings confirm that Tac-MMF remains the gold standard regimen across both age cohorts, utilized in 98.2% of all patients in this study. This high utilization rate of Tac - MMF indicates that older KTRs are exposed to potent immunosuppressive regimens, despite being more susceptible to drug associated complications like infections and malignances due to age related immune dysfunction (22). This observation is consistent with a previous study that analyzed integrated Organ Procurement and Transplantation Network (OPTN)/United Network for Organ Sharing (UNOS) records with Medicare billing claims (22). Here, the authors found a shift towards Tac-based regimens in both younger and older KTRs while the utilization of CsA- and mTORi-based regimens decreased over the analyzed period (**Supplementary Table 3**).

The OS in our cohort was 95.6% and 87.6% at 1- and 5-years, respectively, aligning with previous reports (8, 33). Among the other maintenance regimen evaluated, the highest 5-year cumulative incidence of death was observed in the CsA-MMF and mTORi-MMF groups across both age categories. This finding is consistent with previous studies reporting an increased risk of mortality among KTRs receiving CsA- and mTORi-based therapy (21, 22, 35).

Cause-specific analysis revealed a significantly higher risk of infectious death among older KTRs. Applying standard immunosuppressive regimens to this cohort may increase the risk of over-immunosuppression, contributing to death with a functioning graft (36–39). Furthermore, older KTRs are at a higher risk of developing cardiovascular events, including myocardial infarction, stroke, and heart failure (40). Since immunosuppressive regimens can exacerbate these risks by impairing the regulation of inflammation and oxidative stress, key drivers of vascular disease (41), the implementation of more individualized, tailored immunosuppressive strategies will be crucial for this particularly vulnerable population.

Given that immunosenescence decreases the overall risk of AR in older graft recipients (11), we found at slightly reduced 1-year AR rates in older KTRs when comparing to younger KTRs receiving the same immunosuppressive regimen. These findings are in line with a previous registry analysis (23), which demonstrated lower rejection rates in older KTRs (14% in ≥ 70 years vs. 28% in 18–29 years) under comparable immunosuppression. Notably, the Tac-mTORi combination showed AR rates similar to Tac-MMF, which is also supported by previous reports (42, 43). This finding might open the door for safe CNI-sparing to avoid nephrotoxicity in combination with mTORi.

Previous studies suggested that the lower rejection rates and higher infectious mortality in older patients stem from age-associated immune changes rather than differences in drug metabolism (44). Experimental research has shown that the diminished alloresponse in the elderly is linked to attenuated effector CD4+ T-cell activation, an increased pool of memory T cells, and preserved function of regulatory T cells, suggesting that immune aging modulates the balance between activation and tolerance (38). Moreover, age-related pharmacokinetic alterations, including reduced hepatic first-pass metabolism and altered drug clearance, may increase systemic exposure to immunosuppressive agents (45). These factors underline the importance of individualized dosing and close therapeutic drug monitoring in older KTRs. Similar to a recent study (33) which reported that intensified immunosuppressive regimens are associated with increased infection-related mortality in elderly KTRs, our results provide further evidence that standard immunosuppressive strategies may not be universally applicable to this subgroup. Collectively, these findings advocate for a precision-medicine approach, integrating biological age, frailty status, and pharmacokinetic variability into protocol selection.

Regarding kidney graft function, patients receiving mTORi-MMF presented the lowest mean eGFR at baseline after transplantation and throughout the follow-up period. These findings likely reflect intended CNI avoidance in grafts with poor initial function; however, consistent with previous studies, this group also exhibited a higher risk of AR (46). Longitudinal analysis revealed distinct age-specific eGFR trends: while Tac-MMF provided the most stable kidney function in younger KTRs, a different pattern emerged in the older cohort. Notably, older patients treated with Tac-mTORi showed no significant additional eGFR reduction over time. These results may be suggestive that mTORi-based regimens may offer an individual alternative to Tac-MMF in selected older patients balancing efficacy and tolerability.

The primary strength of our study is based on the utilization of the Scientific Registry of Transplant Recipients (SRTR), a national database that provided a large and diverse cohort of transplant recipients. These data allowed us to evaluate also less common immunosuppressive regimens, such as the tacrolimus–mTORi combination, which are often underrepresented in clinical trials. Furthermore, our study employed a robust variable selection process, incorporating traditional literature-based predictors (1, 5, 21–25) and Random Forest algorithms to identify and control relevant covariates. By specifically focusing on recipients aged ≥ 65 years, we addressed a critical gap in the literature regarding individualized immunosuppression in a rapidly growing and vulnerable patient population.

Despite these strengths, several limitations warrant careful consideration. First, as a retrospective registry analysis, this study is subject to inherent selection and confounding bias. The distribution of regimens was highly imbalanced; specifically, the mTORi-MMF group was significantly smaller than the Tac-MMF group. It is probable that patients in the mTORi-based groups represented a highly selected subgroup, which might have influenced the clinician’s choice of regimen. While we attempted to mitigate this by adjusting for baseline variables selected via literature evidence, univariate analysis, and Random Forest modeling, the SRTR lacks clinical data on frailty, cognitive status, and the specific clinical rationale behind regimen selection. Second, because this study relies on registry data, other clinical parameters were unavailable, including drug-specific side effects, toxicities, and medication adherence related to the immunosuppressive maintenance regimens. Third, the immunosuppressive regimen was defined solely at the therapy received three months after transplantation. Our analysis did not account for therapy “crossover” or modifications during follow-up, which may lead to exposure misclassification. Fourth, although we aimed to identify age-specific effects, the interaction analyses between age and treatment regimens did not reach statistical significance for several primary outcomes. Therefore, the differences observed between immunosuppressive regimens in older KTRs should be interpreted as explorative rather than definitive.

In conclusion, our analysis showed several critical implications of both mTORi- and CNI-based immunosuppressive protocols in older KTRs. As older patients develop immunosenescence, they usually require less aggressive immunosuppression while at the same time being vulnerable to severe adverse effects of immunosuppression including opportunistic infections, malignancies and metabolic toxicities. This is reflected in our results since the older cohort exhibited higher mortality due to infection, cardiovascular disease and malignancy when compared to recipients < 65 years. On the other hand, our findings demonstrated that Tac-mTORi and Tac-MMF regimens exhibited no significant differences when comparing clinical outcomes in both age groups. Therefore, the Tac–mTORi combination may represent an alternative, CNI-sparing strategy in selected older kidney graft recipients. Taken together, our study emphasizes the need for individualized immunosuppressive regimens in older KTRs to optimize clinical outcomes while minimizing the risk of adverse events at the same time calling for further prospective studies.

## Data Availability

The data analyzed in this study is subject to the following licenses/restrictions: The data reported here have been supplied by the Hennepin Healthcare Research Institute (HHRI) as the contractor for the Scientific Registry of Transplant Recipients (SRTR). Requests to access these datasets should be directed to https://srtr.transplant.hrsa.gov/.

## References

[1] Jung CW Jorgensen D Sood P Mehta R Molinari M Hariharan S . Outcomes and factors leading to graft failure in kidney transplants from deceased donors with acute kidney injury-a retrospective cohort study. PloS One. (2021) 16:e0254115. doi: 10.1371/journal.pone.0254115 34437548 PMC8389362

[2] Harambat J Bonthuis M van Stralen KJ Ariceta G Battelino N Bjerre A . Adult height in patients with advanced CKD requiring renal replacement therapy during childhood. Clin J Am Soc Nephrol. (2014) 9:92–9. doi: 10.2215/cjn.00890113 24178977 PMC3878688

[3] Wolfe RA Merion RM Roys EC Port FK . Trends in organ donation and transplantation in the United States, 1998-2007. Am J Transplant. (2009) 9:869–78. doi: 10.1111/j.1600-6143.2009.02564.x 19341412

[4] Fleetwood VA Caliskan Y Rub FAA Axelrod D Lentine KL . Maximizing opportunities for kidney transplantation in older adults. Curr Opin Nephrol Hypertens. (2023) 32:204–11. doi: 10.1097/mnh.0000000000000871 36633323

[5] Wolfe RA Ashby VB Milford EL Ojo AO Ettenger RE Agodoa LY . Comparison of mortality in all patients on dialysis, patients on dialysis awaiting transplantation, and recipients of a first cadaveric transplant. N Engl J Med. (1999) 341:1725–30. doi: 10.1056/nejm199912023412303 10580071

[6] Rao PS Merion RM Ashby VB Port FK Wolfe RA Kayler LK . Renal transplantation in elderly patients older than 70 years of age: results from the Scientific Registry of Transplant Recipients. Transplantation. (2007) 83:1069–74. doi: 10.1097/01.tp.0000259621.56861.31 17452897

[7] Zheng J Cao Y Wang Z Nian Y Guo L Song W . Frailty and prognosis of patients with kidney transplantation: a meta-analysis. BMC Nephrol. (2023) 24:303. doi: 10.1186/s12882-023-03358-0 37833650 PMC10576274

[8] Vanhove T Elias N Safa K Cohen-Bucay A Schold JD Riella LV . Long-term outcome reporting in older kidney transplant recipients and the limitations of conventional survival metrics. Kidney Int Rep. (2022) 7:2397–409. doi: 10.1016/j.ekir.2022.08.010 36531880 PMC9751842

[9] Haugen CE Gross A Chu NM Norman SP Brennan DC Xue QL . Development and validation of an inflammatory-frailty index for kidney transplantation. J Gerontol A Biol Sci Med Sci. (2021) 76:470–7. doi: 10.1093/gerona/glaa167 32619229 PMC7907494

[10] McAdams-DeMarco MA Law A King E Orandi B Salter M Gupta N . Frailty and mortality in kidney transplant recipients. Am J Transplant. (2015) 15:149–54. doi: 10.1111/ajt.12992 25359393 PMC4332809

[11] Tullius SG Milford E . Kidney allocation and the aging immune response. N Engl J Med. (2011) 364:1369–70. doi: 10.1056/nejmc1103007 21410395

[12] Memmos E Lioulios G Antoniadis N Fylaktou A Stangou M . Immunosenescence in kidney transplant recipients. Transplantation. (2025) 109(12):pe675–81. doi: 10.1097/tp.0000000000005484 40653612

[13] Blosser CD Huverserian A Bloom RD Abt PD Goral S Thomasson A . Age, exclusion criteria, and generalizability of randomized trials enrolling kidney transplant recipients. Transplantation. (2011) 91:858–63. doi: 10.1097/tp.0b013e31820f42d9 21325996 PMC3462443

[14] Danovitch GM Gill J Bunnapradist S . Immunosuppression of the elderly kidney transplant recipient. Transplantation. (2007) 84:285–91. doi: 10.1097/01.tp.0000275423.69689.dc 17700150

[15] Older adults "Chronic disease indicators": US center for disease control and prevention. Available online at: https://www.cdc.gov/cdi/indicator-definitions/older-adults.html (Accessed February 15, 2026).

[16] Marino ML Rosa AC Finocchietti M Bellini A Poggi FR Massari M . Temporal and spatial variability of immunosuppressive therapies in transplant patients: an observational study in Italy. Front Transplant. (2022) 1:1060621. doi: 10.3389/frtra.2022.1060621 38994384 PMC11235261

[17] Noble J Leon J Del Bello A Anglicheau D Blancho G Ville S . Belatacept in kidney transplantation: reflecting on the past, shaping the future. Transpl Int. (2025) 38:14412. doi: 10.3389/ti.2025.14412 40463417 PMC12129774

[18] Kidney Disease: Improving Global Outcomes Transplant Work G . KDIGO clinical practice guideline for the care of kidney transplant recipients. Am J Transplant. (2009) 9:S1–S155. doi: 10.1111/j.1600-6143.2009.02834.x 19845597

[19] Bellini A Finocchietti M Rosa AC Nordio M Ferroni E Massari M . Effectiveness and safety of immunosuppressive regimens used as maintenance therapy in kidney transplantation: the CESIT study. PloS One. (2024) 19:e0295205. doi: 10.1371/journal.pone.0295205 38165971 PMC10760756

[20] Inker LA Eneanya ND Coresh J Tighiouart H Wang D Sang Y . New creatinine- and cystatin C-based equations to estimate GFR without race. N Engl J Med. (2021) 385:1737–49. doi: 10.1056/nejmoa2102953 34554658 PMC8822996

[21] Lemoine M Titeca Beauport D Lobbedez T Choukroun G Hurault de Ligny B Hazzan M . Risk factors for early graft failure and death after kidney transplantation in recipients older than 70 years. Kidney Int Rep. (2019) 4:656–66. doi: 10.1016/j.ekir.2019.01.014 31080920 PMC6506713

[22] Lentine KL Cheungpasitporn W Xiao H McAdams-DeMarco M Lam NN Segev DL . Immunosuppression regimen use and outcomes in older and younger adult kidney transplant recipients: a national registry analysis. Transplantation. (2021) 105:1840–9. doi: 10.1097/tp.0000000000003547 33214534 PMC10576532

[23] Tullius SG Tran H Guleria I Malek SK Tilney NL Milford E . The combination of donor and recipient age is critical in determining host immunoresponsiveness and renal transplant outcome. Ann Surg. (2010) 252:662–74. doi: 10.1097/sla.0b013e3181f65c7d 20881773

[24] Foroutan F Friesen EL Clark KE Motaghi S Zyla R Lee Y . Risk factors for 1-year graft loss after kidney transplantation: systematic review and meta-analysis. Clin J Am Soc Nephrol. (2019) 14:1642–50. doi: 10.2215/CJN.05560519 PMC6832056 31540931

[25] Kaboré R Haller MC Harambat J Heinze G Leffondré K . Risk prediction models for graft failure in kidney transplantation: a systematic review. Nephrol Dial Transplant. (2017) 32:ii68–76. doi: 10.1186/s12874-024-02445-6 28206633

[26] Berkowitz M Altman RM Loughin TM . Random forests for survival data: which methods work best and under what conditions? Int J Biostat. (2024) 20:315–45. doi: 10.1515/ijb-2023-0056 38656274 PMC11661562

[27] Pickett KL Suresh K Campbell KR Davis S Juarez-Colunga E . Random survival forests for dynamic predictions of a time-to-event outcome using a longitudinal biomarker. BMC Med Res Methodol. (2021) 21:216. doi: 10.1186/s12874-021-01375-x 34657597 PMC8520610

[28] Qiu X Gao J Yang J Hu J Hu W Kong L . A comparison study of machine learning (random survival forest) and classic statistic (Cox proportional hazards) for predicting progression in high-grade glioma after proton and carbon ion radiotherapy. Front Oncol. (2020) 10:2020. doi: 10.3389/fonc.2020.551420 33194609 PMC7662123

[29] Li J Scheike TH Zhang MJ . Checking Fine and Gray subdistribution hazards model with cumulative sums of residuals. Lifetime Data Anal. (2015) 21:197–217. doi: 10.1007/s10985-014-9313-9 25421251 PMC4386671

[30] Lentine KL Smith JM Lyden GR Miller JM Booker SE Dolan TG . OPTN/SRTR 2023 annual data report: kidney. Am J Transplant. (2025) 25:S22–S137. doi: 10.1016/j.ajt.2025.01.020 39947805 PMC12414513

[31] Hart A Smith JM Skeans MA Gustafson SK Wilk AR Castro S . OPTN/SRTR 2018 annual data report: kidney. Am J Transplant. (2020) 20:20–130. doi: 10.1111/ajt.15672 31898417

[32] Sanders JF de Boer SE Jonker J Bemelman FJ Betjes MGH de Vries APJ . Immunosuppression in older kidney transplant recipients: a randomized controlled trial. J Am Soc Nephrol. (2026) 37:814–24. doi: 10.1681/ASN.0000000924 PMC13065175 41201871

[33] Hamdy MY El Aggan H El Gohary I Halawa A . Renal transplant in elderly end-stage renal disease patients: impact of comorbidities and posttransplant adverse events on outcomes. Exp Clin Transplant. (2024) 22:93–102. doi: 10.6002/ect.2023.0301 38511980

[34] Pravisani R Isola M Baccarani U Crestale S Tulissi P Vallone C . Impact of kidney transplant morbidity on elderly recipients' outcomes. Aging Clin Exp Res. (2021) 33:625–33. doi: 10.1007/s40520-020-01558-4 32323169

[35] Huang E Poommipanit N Sampaio MS Kuo HT Reddy P Gritsch HA . Intermediate-term outcomes associated with kidney transplantation in recipients 80 years and older: an analysis of the OPTN/UNOS database. Transplantation. (2010) 90:974–9. doi: 10.1097/tp.0b013e3181f5c3bf 20814353

[36] Alshaer I Hung RKY Basu S Goldet G Jones G Harber M . Older kidney transplant patients are over immunosuppressed using standard protocols with differential sex-based complications. J Transplant. (2025) 2025:5547629. doi: 10.1155/joot/5547629 41127051 PMC12539993

[37] Betjes MG . Immune cell dysfunction and inflammation in end-stage renal disease. Nat Rev Nephrol. (2013) 9:255–65. doi: 10.1038/nrneph.2013.44 23507826

[38] So S Au EHK Lim WH Lee VWS Wong G . Factors influencing long-term patient and allograft outcomes in elderly kidney transplant recipients. Kidney Int Rep. (2021) 6:727–36. doi: 10.1016/j.ekir.2020.11.035 33732987 PMC7938063

[39] Adani GL Baccarani U Crestale S Pravisani R Isola M Tulissi P . Kidney transplantation in elderly recipients: a single-center experience. Transplant Proc. (2019) 51:132–5. doi: 10.1016/j.transproceed.2018.04.081 30661894

[40] Rangaswami J Mathew RO Parasuraman R Tantisattamo E Lubetzky M Rao S . Cardiovascular disease in the kidney transplant recipient: epidemiology, diagnosis and management strategies. Nephrol Dial Transplant. (2019) 34:760–73. doi: 10.1093/ndt/gfz053 30984976

[41] Zhazykbayeva S Pabel S Mügge A Sossalla S Hamdani N . The molecular mechanisms associated with the physiological responses to inflammation and oxidative stress in cardiovascular diseases. Biophys Rev. (2020) 12:947–68. doi: 10.1007/s12551-020-00742-0 32691301 PMC7429613

[42] Ciancio G Burke GW Gaynor JJ Ruiz P Roth D Kupin W . A randomized long-term trial of tacrolimus/sirolimus versus tacrolimums/mycophenolate versus cyclosporine/sirolimus in renal transplantation: three-year analysis. Transplantation. (2006) 81:845–52. doi: 10.1097/01.tp.0000203894.53714.27 16570006

[43] Zou ZY Dai LR Hou YB Yu CZ Chen RJ Chen YY . Sirolimus in combination with low-dose extended-release tacrolimus in kidney transplant recipients. Front Med (Lausanne). (2023) 10:1281939. doi: 10.3389/fmed.2023.1281939 38105889 PMC10722907

[44] Tang JT de Winter BC Hesselink DA Sombogaard F Wang LL van Gelder T . The pharmacokinetics and pharmacodynamics of mycophenolate mofetil in younger and elderly renal transplant recipients. Br J Clin Pharmacol. (2017) 83:812–22. doi: 10.1111/bcp.13154 27753146 PMC5346874

[45] Jacobson PA Schladt D Oetting WS Leduc R Guan W Matas AJ . Lower calcineurin inhibitor doses in older compared to younger kidney transplant recipients yield similar troughs. Am J Transplant. (2012) 12:3326–36. doi: 10.1111/j.1600-6143.2012.04232.x 22947444 PMC3513646

[46] Zeng J Zhong Q Feng X Li L Feng S Fan Y . Conversion from calcineurin inhibitors to mammalian target of rapamycin inhibitors in kidney transplant recipients: a systematic review and meta-analysis of randomized controlled trials. Front Immunol. (2021) 12:663602. doi: 10.3389/fimmu.2021.663602 34539621 PMC8446650

