# Supplementary material for: Age-specific impact of different immunosuppressive regimens on kidney transplant outcomes: clinical evidence from the scientific registry of transplant recipients
Source: Front Nephrol. 2026 Aug 14;6:1886887. doi: 10.3389/fneph.2026.1886887 (PMC13521798; doi:10.3389/fneph.2026.1886887)
Supplement: Supplementary file 1 [file Table1.docx]

**Tables supplementals**

**Table 1S:** Survival probability (including death after graft loss) at 2 and 5 years after transplantation stratified by age and immunosuppressive regimen

|  | **Tac - MMF** | **Tac - mTORi** | **CsA - MMF** | **mTORi - MMF** |
| --- | --- | --- | --- | --- |
| *At 2 years* |  |  |  |  |
| 18 - 64 years | 97.0% | 96.9% | 96.8% | 91.3% |
| ≥ 65 years | 91.6% | 93.5% | 88.0% | 83.5% |
| *At 5 years* |  |  |  |  |
| 18 - 64 years | 90.8% | 90.9% | 88.6% | 79.4% |
| ≥ 65 years | 74.8% | 79.0% | 68.0% | 61.3% |

**Table 2S:** Univariable analysis of the endpoint all-cause of death, proportional hazard ratio model (n = 111,975)

|  | **HR** | **95% CI** | **P value** |
| --- | --- | --- | --- |
| Gender male | 1.202 | 1.159 – 1.247 | < 0.001 |
| Age 18 - 64 years | 0.333 | 0.321 – 0.345 | < 0.001 |
| Weight | 1.004 | 1.004 – 1.005 | < 0.001 |
| BMI | 1.020 | 1.017 – 1.024 | < 0.001 |
| Dialysis prior transplantation yes | 1.957 | 1.857 – 2.063 | <0.001 |
| Previous Diabetes yes | 2.521 | 2.434 – 2.611 | <0.001 |
| Previous Malignancy yes | 1.544 | 1.350 – 1.767 | <0.001 |
| Time on wait list | 1.006 | 1.005 – 1.007 | <0.001 |
| Number of HLA mismatches | 1.084 | 1.071 – 1.096 | <0.001 |
| Cold ischemic times |  |  |  |
| 0 hours | 1 |  |  |
| 1 – 6 hours | 1.243 | 1.166 – 1.326 | <0.001 |
| > 6 – 12 hours | 2.011 | 1.890 – 2.138 | <0.001 |
| > 12 – 18 hours | 2.326 | 2.191 – 2.468 | <0.001 |
| > 18 – 24 hours | 2.551 | 2.394 – 2.718 | <0.001 |
| > 24 hours | 2.547 | 2.388 – 2.716 | <0.001 |
| Donor age in years | 1.023 | 1.021 – 1.024 | <0.001 |
| Donor gender female | 0.965 | 0.932 – 0.998 | 0.0485 |
| Donor type | 2.228 | 2.142 – 2.317 | <0.001 |
| living n | 1 |  |  |
| deceased n | 1.872 | 1.796 – 1.952 | <0.001 |
| deceased expanded criteria donor n | 3.810 | 3.625 – 4.004 | <0.001 |
| Expanded criteria donors | 2.650 | 2.542 – 2.762 | <0.001 |
| Cretinine > 1.5 mg/dl | 1.074 | 1.022 – 1.129 | <0.001 |
| Induction therapy yes | 0.920 | 0.866 – 0.977 | 0.007 |
| Steroid induction yes | 0.998 | 0.962 – 1.036 | 0.930 |
| Steroid maintenance yes | 1.092 | 1.052 – 1.134 | <0.001 |
| Immunotherapy |  |  |  |
| Tac-MMF | 1 |  |  |
| Tac-mTORi | 0.858 | 0.676 – 1.090 | 0.210 |
| CsA-MMF | 1.150 | 1.015 – 1.301 | 0.028 |
| mTORi-MMF | 1.958 | 1.544 – 2.482 | <0.001 |

**Table 3S:** Cumulative incidence of graft loss and death with functioning graft at 2 and 5 years after transplantation stratified by age and immunosuppressive regimen

|  | **Tac - MMF** | | **Tac - mTORi** | | **CsA - MMF** | | **mTORi - MMF** | |
| --- | --- | --- | --- | --- | --- | --- | --- | --- |
|  | **Graft loss** | ***Death** | **Graft loss** | ***Death** | **Graft loss** | ***Death** | **Graft loss** | ***Death** |
| *At 2 years* |  |  |  |  |  |  |  |  |
| 18 - 64 years | 3.4% | 2.5% | 2.6% | 2.8% | 6.9% | 2.6% | 9.6% | 7.7% |
| ≥ 65 years | 4.1% | 6.8% | 1.0% | 6.5% | 7.3% | 8.2% | 8.4% | 13.4% |
| *At 5 years* |  |  |  |  |  |  |  |  |
| 18 - 64 years | 9.5% | 7.0% | 9.8% | 7.9% | 14.4% | 8.2% | 20.9% | 15.3% |
| ≥ 65 years | 8.3% | 20.3% | 7.1% | 18% | 11.7% | 24.6% | 21.3% | 30.2% |

*Death with functioning graft

**Table 4S:** Percentage of acute rejection stratified by age and immunosuppressive regimen (n = 104,788)

|  | **Age 18 - 64 years (N=83,312)** | **Age ≥ 65 years (N=21,476)** |
| --- | --- | --- |
| **TAC - MMF** |  |  |
| Not n (%) | 75,374 (92.2%) | 19,604 (93.2%) |
| Yes n (%) | 6,359 (7.8%) | 1,432 (6.8%) |
| **TAC - mTORi** |  |  |
| Not n (%) | 357 (88.8%) | 86 (91.5%) |
| Yes n (%) | 45 (11.2%) | 8 (8.5%) |
| **CsA - MMF** |  |  |
| Not n (%) | 795 (85.1%) | 247 (90.1%) |
| Yes n (%) | 139 (14.9%) | 27 (9.9%) |
| **mTORi - MMF** |  |  |
| Not n (%) | 202 (83.1%) | 61 (84.7%) |
| Yes n (%) | 41 (16.9%) | 11 (15.3%) |

**Table 5S: GFR values during the first 5 years after kidney transplantation**

| **Therapy** | **Time** | **GFR mean** | **GFR sd** |
| --- | --- | --- | --- |
| Age. 18 - 64 years |  |  |  |
| Tac - MMF | 1 month | 43.95 | 28.97 |
| Tac - MMF | 6 months | 63.01 | 20.54 |
| Tac - MMF | 1 year | 63.35 | 20.86 |
| Tac - MMF | 2 years | 63.85 | 21.51 |
| Tac - MMF | 3 years | 63.68 | 22.13 |
| Tac - MMF | 4 years | 63.26 | 22.58 |
| Tac - MMF | 5 years | 62.96 | 23.02 |
| Tac - mTORi | 1 month | 46.46 | 27.95 |
| Tac - mTORi | 6 months | 64.05 | 21.26 |
| Tac - mTORi | 1 year | 62.88 | 21.03 |
| Tac - mTORi | 2 years | 60.50 | 21.99 |
| Tac - mTORi | 3 years | 62.44 | 23.01 |
| Tac - mTORi | 4 years | 60.02 | 23.32 |
| Tac - mTORi | 5 years | 58.81 | 23.82 |
| CsA - MMF | 1 month | 43.92 | 27.66 |
| CsA - MMF | 6 months | 59.10 | 20.81 |
| CsA - MMF | 1 year | 56.60 | 20.27 |
| CsA - MMF | 2 years | 56.35 | 21.48 |
| CsA - MMF | 3 years | 56.27 | 22.43 |
| CsA - MMF | 4 years | 55.11 | 22.06 |
| CsA - MMF | 5 years | 54.70 | 22.46 |
| mTORi - MMF | 1 month | 29.41 | 25.27 |
| mTORi - MMF | 6 months | 58.51 | 22.86 |
| mTORi - MMF | 1 year | 55.06 | 23.93 |
| mTORi - MMF | 2 years | 51.49 | 22.99 |
| mTORi - MMF | 3 years | 51.47 | 23.49 |
| mTORi - MMF | 4 years | 52.34 | 23.33 |
| mTORi - MMF | 5 years | 52.16 | 24.42 |
| Age ≥ 65 years |  |  |  |
| Tac - MMF | 1 month | 39.52 | 26.63 |
| Tac - MMF | 6 months | 57.55 | 19.01 |
| Tac - MMF | 1 year | 57.62 | 19.54 |
| Tac - MMF | 2 years | 58.25 | 19.82 |
| Tac - MMF | 3 years | 58.32 | 20.49 |
| Tac - MMF | 4 years | 57.97 | 20.76 |
| Tac - MMF | 5 years | 58.20 | 21.48 |
| Tac - mTORi | 1 month | 40.45 | 24.37 |
| Tac - mTORi | 6 months | 59.01 | 17.30 |
| Tac - mTORi | 1 year | 58.75 | 18.17 |
| Tac - mTORi | 2 years | 57.37 | 19.51 |
| Tac - mTORi | 3 years | 57.58 | 19.62 |
| Tac - mTORi | 4 years | 57.29 | 20.91 |
| Tac - mTORi | 5 years | 60.66 | 18.53 |
| CsA - MMF | 1 month | 38.58 | 25.70 |
| CsA - MMF | 6 months | 56.05 | 19.03 |
| CsA - MMF | 1 year | 56.68 | 20.24 |
| CsA - MMF | 2 years | 56.33 | 19.53 |
| CsA - MMF | 3 years | 54.29 | 19.67 |
| CsA - MMF | 4 years | 55.60 | 21.41 |
| CsA - MMF | 5 years | 54.78 | 22.06 |
| mTORi - MMF | 1 month | 25.31 | 19.56 |
| mTORi - MMF | 6 months | 47.26 | 14.82 |
| mTORi - MMF | 1 year | 47.82 | 16.55 |
| mTORi - MMF | 2 years | 48.36 | 19.19 |
| mTORi - MMF | 3 years | 46.98 | 19.55 |
| mTORi - MMF | 4 years | 47.17 | 19.69 |
| mTORi - MMF | 5 years | 48.67 | 24.33 |

**Figures supplementals**

**Figure 1S:** Random forest, variable selection

**Figure 2S:** Stacked cumulative incidence of graft loss including competing risk estimates stratified by age and maintenance immunosuppressive regimen

This cumulative incidence function plots the probability of being in the state “alive with functioning graft”, “death with functioning graft” or “graft failure” throughout the follow-up period.

**Figure 3S: Evolution of maintenance immunosupresive regimens stratified by year of kidney transplantation**
